# Neutrophil extracellular traps in pregnancy complications

**DOI:** 10.3389/fimmu.2026.1798749

**Published:** 2026-04-10

**Authors:** Chunying Wang, Meihua Zhang

**Affiliations:** Key Laboratory of Maternal & Fetal Medicine of National Health Commission of China, Shandong Provincial Maternal and Child Health Care Hospital Affiliated to Qingdao University, Jinan, China

**Keywords:** neutrophil, neutrophil extracellular traps, obstetric drugs, preeclampsia, pregnancy complications

## Abstract

Neutrophil extracellular traps (NETs) are fibrous, web-like structures released by activated neutrophils that consist of decondensed chromatin DNA coated with antimicrobial granular proteins. These structures play a dual role in host defense and pathology by effectively entrapping and eliminating pathogens. Under normal physiological conditions during pregnancy, appropriately regulated NET formation at the maternal–fetal interface functions as a crucial antimicrobial defense mechanism. However, emerging evidence indicates that excessive NET formation or defective clearance is strongly linked to the pathogenesis of several pregnancy complications such as preeclampsia, gestational diabetes mellitus, preterm birth, recurrent pregnancy loss, systemic lupus erythematosus, and obstetric antiphospholipid syndrome. This review systematically examines the regulatory mechanisms and pathophysiological contributions of NETs to these pregnancy complications. This review further explores the potential therapeutic mechanisms of common obstetric medications—including aspirin, metformin, low molecular weight heparin, hydroxychloroquine, and vitamin D-which may exert beneficial effects by suppressing NET formation or enhancing NET clearance.

## 1 Introduction

Neutrophils are key effector cells in host defense against pathogens. In addition to their antimicrobial mechanisms, recent research has highlighted a distinctive fibrous mesh-like chromatin structure called neutrophil extracellular traps (NETs) (1). NETs enable neutrophils to immobilize and capture pathogens and subsequently eliminate bacteria, viruses, fungi, and other microbes via associated antimicrobial components, thus constituting a significant addition to the innate immune defense system (2). Beyond their primary role in microbial clearance, NETs also participate in diverse physiological processes, including immunomodulation, thrombosis, inflammation resolution, and tissue repair. However, NETs act as a double-edged sword, whereby excessive or persistent accumulation *in vivo* may exacerbate inflammatory responses and even induce tissue damage (3).

Pregnancy is a unique physiological state characterized by complex maternal-fetal interactions. The maintenance of immune homeostasis at the maternal-fetal interface is essential for the normal progression of pregnancy. As vital innate immune cells, neutrophils are strongly involved in placental development and the regulation of homeostasis at the maternal-fetal interface (4–6). A growing body of research suggests that during normal pregnancy, finely tuned NET formation under the precise regulation of the maternal-fetal interface microenvironment serves as an essential line of defense against pathogenic infections (6). Circulating factors such as granulocyte colony-stimulating factor (G-CSF), human chorionic gonadotropin (hCG), and estradiol (E2) promote NET generation, whereas progesterone (P4) and vasoactive intestinal peptide (VIP) secreted by trophoblasts inhibit neutrophil activation, thereby preventing excessive NET formation and tissue injury, together forming a dynamically balanced regulatory network (7, 8).

In recent years, increasing evidence has demonstrated that dysregulation of NET homeostasis is closely associated with the pathogenesis and progression of various pregnancy complications. Aberrant upregulation of NET formation or compromised clearance has been reported in common obstetric disorders, including preeclampsia (PE) (9), gestational diabetes mellitus (GDM) (10), preterm birth (PTB) (11), and recurrent pregnancy loss (RPL) (12) as well as in pregnancies complicated by autoimmune diseases such as systemic lupus erythematosus (SLE) (13) and antiphospholipid syndrome (APS) (14). Excessive NETs disrupt maternal-fetal interface homeostasis through multiple mechanisms, including the induction of inflammatory responses, impairment of vascular endothelium and placental tissues, interference with spiral artery remodeling, and promotion of thrombogenesis, ultimately leading to adverse pregnancy outcomes (APOs) (14–16). Given the involvement of NETs in the pathophysiology of obstetric disorders, targeting NET generation may represent a potential therapeutic strategy. Commonly used clinical agents in obstetrics, such as aspirin, metformin, low molecular weight heparin (LMWH), hydroxychloroquine (HCQ) and vitamin D, have been shown to exert potential therapeutic effects by inhibiting NET formation or promoting NET clearance (17–21); however, their specific regulatory mechanisms in pregnancy complications require further investigation.

This review synthesizes the existing research to elucidate the causes, regulatory mechanisms, and pathophysiological roles of NETs in various pregnancy complications. Additionally, it examines potential novel mechanisms by which commonly used obstetric medications modulate NET homeostasis, offering new theoretical insights and research avenues for diagnosing and managing these conditions.

## 2 Introduction to neutrophil extracellular traps

### 2.1 Neutrophil

Neutrophils are the most abundant leukocytes in human blood. As a core component of the innate immune system, they form the first line of defense against pathogens (22). Neutrophils are derived from the bone marrow and then enter the circulatory system. Upon pathogen invasion, they are recruited to sites of infection or inflammation, where they transmigrate across the vascular endothelium through a multistep process involving rolling, adhesion, and crawling to eliminate microbes (23). Upon encountering invading pathogens, neutrophils exert their cytotoxic effects through two main mechanisms: phagocytosis, which involves the internalization and degradation of pathogens within phagolysosomes via proteolytic enzymes, antimicrobial proteins, and reactive oxygen species (ROS); and degranulation, whereby antimicrobial factors are released directly into the extracellular space to neutralize pathogens (24). However, in 2007, Brinkmann et al. reported a novel antimicrobial mechanism in neutrophils termed NETosis, which involves the release of NETs to capture and kill pathogens. Classically, neutrophils primarily die through apoptosis, necrosis, or the highly regulated necroptosis, whereas NETosis is a cell death program that is mechanistically distinct from apoptosis and necrosis (25).

Not all neutrophils exhibit identical functional profiles; however, compared with conventional neutrophils, low-density granulocytes (LDGs) are a heterogeneous neutrophil subset isolated in the low-density fraction of peripheral blood mononuclear cells that possess unique proinflammatory and functional characteristics, with markedly stronger proinflammatory potential, including enhanced secretion of proinflammatory cytokines and ROS. Notably, LDGs exhibit increased NET-forming activity and more easily activated to release NETs (26).

### 2.2 Neutrophil extracellular traps

NETs are fibrous, web-like structures consisting of decondensed chromatin fibers decorated with granular proteins, such as myeloperoxidase (MPO), neutrophil elastase (NE), and cathepsin G (27). Studies have demonstrated that various infectious and sterile stimuli can trigger NET formation, including bacteria (28), viruses (28), fungi (29), parasites (30), interleukins (31), tumor necrosis factor α (TNF-α) (32), placental micro-debris (33), activated platelets (34), cholesterol crystals (35), monosodium uric acid (36), autoantibodies (37), and complements (38). Histone citrullination catalyzed by peptidylarginine deiminase 4 (PAD4) contributes to NET formation (39). However, the dependency of NET formation on histone citrullination remains highly controversial, particularly in response to phorbol myristate acetate (PMA) stimulation. For instance, Li et al. (40) and Holmes et al. (41) reported detectable levels of histone citrullination in PMA-activated murine and human neutrophils, respectively (40, 41). In contrast, Neeli and Radic (43) and König and Andrade (42) failed to detect citrullinated histone H3 in PMA-stimulated human neutrophils at the 2-hour time point by Western blotting (42, 43). Collectively, these conflicting results indicate that PAD4-mediated citrullination is not absolutely necessary for PMA-induced NET formation. In addition, NET formation involves ROS production and the activation of MPO and NE (44), which promote histone degradation, chromatin decondensation, and the release of DNA and antimicrobial molecules (45).

### 2.3 NETosis pathways

**Figure 1:**
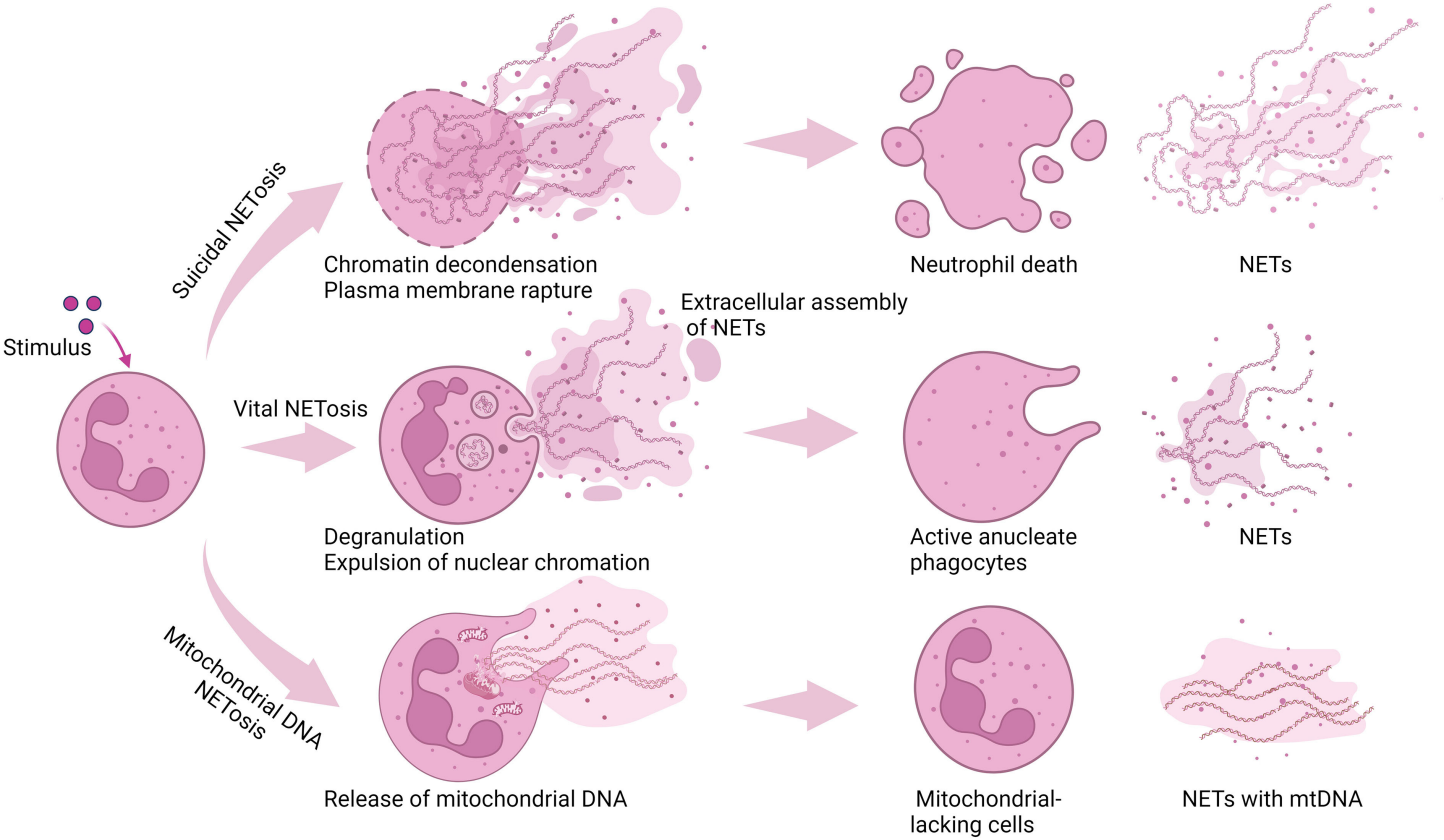
NET formation pathways. Suicidal NETosis involves a stepwise cellular process: nuclear lobulation, nuclear membrane breakdown, loss of cell polarity, chromatin decondensation, and ultimately, plasma membrane rupture. In contrast, vital NETosis is a non-lethal pathway that mediates chromatin extrusion and granular protein release without causing cell death. Following the extracellular assembly of NET components, the resulting anucleate phagocytes remain metabolically active and retain functional capabilities for microbial phagocytosis and chemotaxis. An alternative pathway is mtDNA-driven NETosis: this noncell death-dependent process induces NETs via mitochondrial (not nuclear) DNA, which relies on glycolysis-derived ATP for the cytoskeletal rearrangements essential for mtDNA release and degranulation. Figure created with BioRender.com.

NET formation occurs through two primary pathways (**Figure 1**). The first, known as suicidal NETosis (or lytic NETosis), relies on NADPH oxidase activity. Upon neutrophil stimulation, calcium is released from the endoplasmic reticulum, leading to the activation of protein kinase C (PKC). The activated NADPH oxidase complex then promotes increased levels of ROS, which in turn triggers the degradation of cytoplasmic granules containing MPO and NE (46). NE and MPO subsequently cooperate with PAD4 to induce citrullination of histone H3 (40). This modification facilitates nuclear chromatin decondensation, followed by the rupture of both the nuclear envelope and the plasma membrane, ultimately resulting in the release of NETs into the extracellular space (47). This pathway can be induced by antibodies, microbes, cholesterol crystals, TNFα or IL-6/8 (48, 49). Unlike the suicidal pathway, vital NETosis (also known as non-lytic NETosis) is independent of the activity of NADPH oxidase and can be induced by activated platelets, specific microbes, or the calcium ionophore A23187 (48). Additionally, vital NETosis is a non-lytic process that does not induce cell death. Upon neutrophil activation, calcium influx into cells occurs via small conductance potassium channel member 3, and elevated intracellular Ca^2+^ levels activate PAD4, thereby promoting the citrullination of histone H3 (50). Neutrophils then extrude chromatin and granular components into the extracellular space, where they assemble into NETs, leaving behind an active nuclear cytoplast that retains its capacity for phagocytosis and chemotaxis (51, 52). An alternative mechanism involves mitochondrial DNA-driven NETosis, a pathway characterized by the induction of NETs through the release of mitochondrial DNA, rather than nuclear DNA, in response to specific stimuli such as complement component 5a and lipopolysaccharide (53). This process occurs independently of cell death but depends on ATP generated through glycolysis to power structural reorganizations of the microtubule network and filamentous actin. These cytoskeletal rearrangements are indispensable for both mitochondrial DNA release and degranulation (54).

### 2.4 Clearance of NETs

Efficient clearance of NETs is critical for maintaining immune homeostasis. This process is a complex biological event involving multiple steps and various cell types. To date, NET clearance is known to occur primarily through two mechanisms: nuclease-mediated backbone degradation and phagocyte-mediated fragment removal (55). In the extracellular compartment, the core event is the specific hydrolysis of the DNA backbone of NETs by nucleases, which largely depends on the coordinated action of deoxyribonuclease (DNase) and three prime repair exonuclease (TREX) families (56). The DNase family comprises two subfamilies: DNase I (including DNase I, DNase1L1, DNase1L2, and DNase1L3) and DNase II (including DNase II α, DNase II β, and L-DNase II). Although these DNase subfamilies differ in their biochemical properties, their functions partially overlap. Both subfamilies function by hydrolyzing the phosphodiester bonds of DNA molecules and serve as key enzymes in maintaining low levels of circulating cell-free DNA (56, 57). Among them, the degradation of DNA by DNase I and DNase1L3 represents the rate-limiting step in NET accumulation. These two enzymes can efficiently eliminate intravascular NETs under pathological conditions such as sepsis or sterile neutrophilia (56). In contrast, DNase II is predominantly localized in the lysosomes of various cells, including macrophages, and its core function is to degrade exogenous DNA internalized by macrophages, particularly DNA fragments derived from apoptotic cells (58). In addition to the DNase family, TREX1 and TREX2 act as important supplementary enzymes in NET clearance. A key advantage of these nucleases is their ability to degrade oxidized DNA that is resistant to DNase I and DNase II (56). Following pretreatment of NETs with extracellular nucleases, macrophages take up NET fragments via macropinocytosis and endocytosis. After phagocytosis, NETs are ultimately degraded within lysosomes (59).

### 2.5 Physiological role of NETs

**Figure 2:**
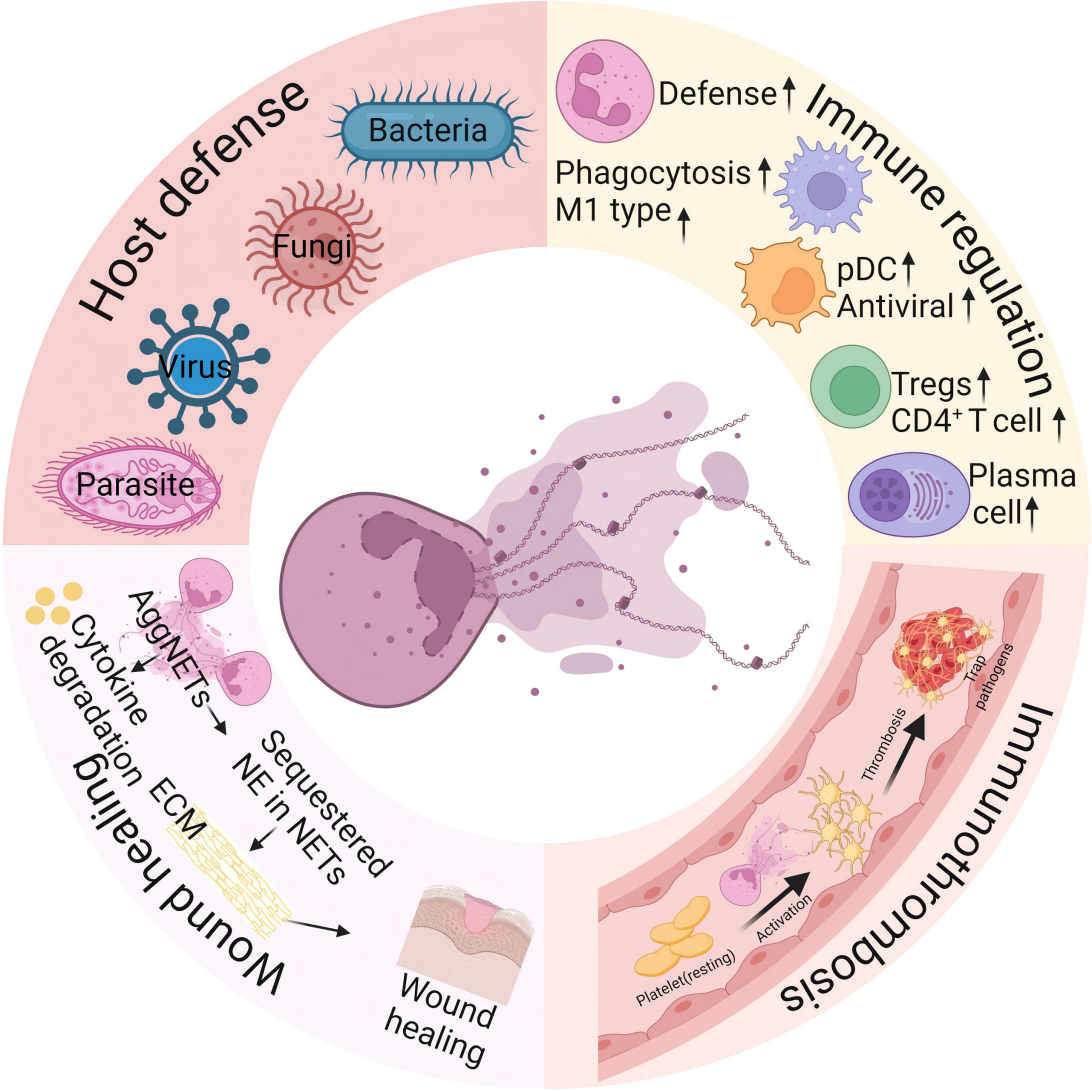
Role of NETs in health. NETs are critical for maintaining homeostasis and functions as follows: (1) host defense via pathogen entrapment and immobilization; (2) immune regulation by boosting neutrophil defense, promoting macrophage polarization, facilitating DC differentiation, and supporting CD4^+^ T/B-cell activation; (3) immunothrombosis through FXII activation, VWF binding, and histone H3/H4-mediated platelet activation; (4) wound healing via AggNET-driven proinflammatory cytokine degradation and NE sequestration to protect the extracellular matrix from proteolysis, thus resolving inflammation and accelerating repair. Figure created with BioRender.com.

The core physiological function of NETs is to restrict pathogen dissemination and directly eliminate microorganisms including bacteria, fungi, viruses, and parasites, thereby aiding the host in combating infections (60). Beyond their well-established function in innate immunity, NETs are critically involved in a variety of physiological processes (**Figure 2**). For instance, they increase neutrophil-mediated defense (61), induce macrophage polarization (62), facilitate the differentiation of dendritic cells (DCs) (63, 64), and contribute to the activation of CD4^+^ T cells and B cells (65, 66), underscoring their broad immunomodulatory functions. Moreover, NETs participate in immunothrombosis by activating coagulation factor XII (67), binding von Willebrand factor (VWF) (68), and promoting platelet activation via histones H3 and H4 (69, 70). Moreover, aggregated NETs can inhibit extracellular matrix proteolysis by degrading proinflammatory cytokines and sequestering NE, thereby facilitating inflammation resolution and tissue repair (71–73).

### 2.6 Controversial role of NETs

While NETs have essential protective effects in physiological processes, emerging evidence has revealed their controversial role in the pathogenesis of various diseases. Excessive NET formation or impaired clearance can disrupt immune homeostasis and trigger exacerbated inflammation and tissue damage, thereby contributing to the development and progression of multiple pathological conditions, including infectious diseases, metabolic disorders, autoimmune diseases, and cancer (74). In infectious diseases, NETs initially act to trap and eliminate invading pathogens (2, 75), but sustained inflammatory stimulation leads to excessive NET release, which aggravates tissue injury and systemic inflammation (76, 77). Neutrophils from diabetic patients exhibit increased susceptibility to NETosis (78), and elevated NET markers are correlated with disease severity and complications (79). In metabolic dysfunction-associated steatotic liver disease, free fatty acids and cholesterol crystals induce excessive NET formation, promoting chronic hepatic inflammation and progression to hepatocellular carcinoma (80, 81). In rheumatoid arthritis (RA), NETs release citrullinated histones that induce ACPA production, exacerbating synovial inflammation (82–84). In SLE, impaired NET clearance leads to the accumulation of NET remnants, triggering complement activation and autoantibody production (85, 86). In cancer, NETs primarily exert protumorigenic effects by promoting tumor cell proliferation, metastasis, and immunosuppression (87, 88). Notably, the pathogenic role of NETs is context dependent, with specific mechanisms varying across diseases. Targeting NET formation or clearance has emerged as a potential therapeutic strategy, highlighting the importance of further exploring their regulatory networks in disease progression.

## 3 NETs in normal pregnancy

Pregnancy refers to the entire process starting from oocyte fertilization, followed by blastocyst implantation in the uterine cavity, development into an embryo and subsequent fetus, and culminating in the delivery of a mature fetus from the maternal body (89). As a unique physiological state involving maternal-fetal interaction, maintaining the balance of local and systemic immunity is crucial for the progression of normal pregnancy (90). As a key component of the innate immune system, neutrophils are dynamically regulated during normal pregnancy and participate in maternal adaptive regulation of pregnancy through multiple mechanisms, including the formation of NETs (91). Abnormal neutrophil function and dysregulated NET formation have been linked to various pregnancy complications, such as PE, GDM, and RPL (92). In comparison with those nonpregnant women, neutrophils during normal pregnancy are modulated by multiple factors to increase NETosis and NET release, a response that progressively increases as gestation advances. The modulation of NET formation during normal pregnancy (first trimester: 1–12 weeks, second trimester: 13–27 weeks, and third trimester: 28 weeks to term) is tightly regulated by pregnancy-related hormones and cytokines with stage-specific effects. Circulating G-CSF levels increase during pregnancy, and G-CSF acts as a core pro-NETotic factor that drives NET formation. hCG exerts a pro-NETotic effect primarily during the first trimester, whereas E2 functions as a pro-NETotic hormone during late pregnancy. In contrast, P4, which peaks during the third trimester, antagonizes the pro-NETotic effects of E2 and G-CSF by blocking NE translocation from the cytoplasm to the nucleus, thus confining neutrophils to a primed pro-NETotic state without full NETosis. This hormonal regulation exerts key physiological effects: moderate pro-NETotic effects of G-CSF, hCG and E2 enhance antimicrobial defense at the maternal-fetal interface via NETs to fend off intrauterine infections. The inhibitory effect of P4 prevents excessive NET release-induced endothelial and placental damage, safeguarding maternal-fetal immune homeostasis, and the primed state maintained by P4 enables rapid NET formation in response to pathogenic stimuli when needed (7). Furthermore, trophoblast-derived VIP suppresses ROS production in neutrophils, thereby inhibiting NET formation. This VIP-mediated dual regulation of neutrophils maintains maternal-fetal immune homeostasis at the placental interface, thus safeguarding the normal progression of early pregnancy (8). As key neutrophil effector products, NETs contribute to maternal-fetal interface defense during normal pregnancy. However, their precise roles in placental development and maternal adaptation remain unclear. A well-balanced NET formation is essential for normal pregnancy, but NET dysregulation leads to adverse outcomes.

## 4 NETs in pregnancy complications

**Figure 3:**
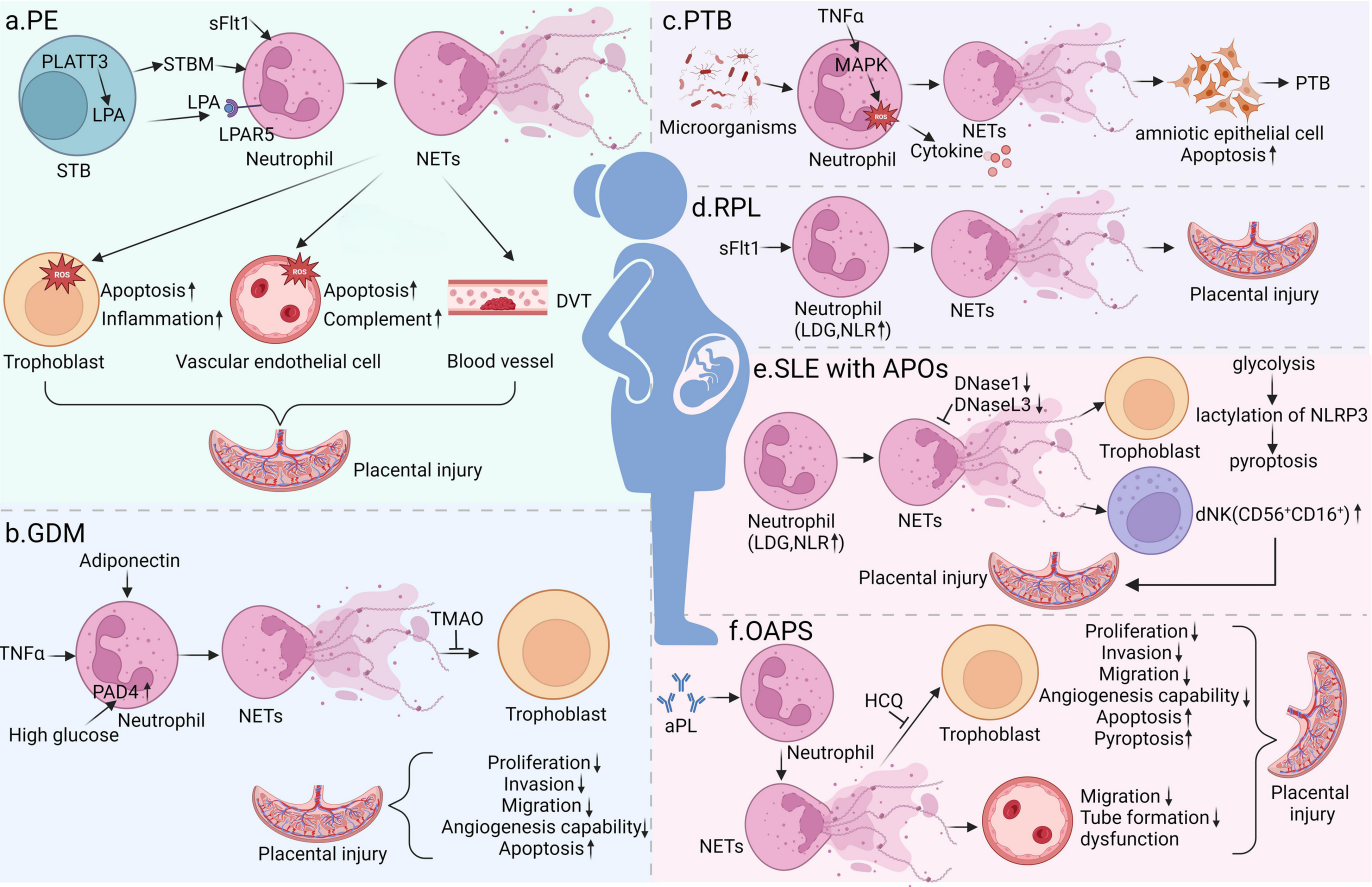
NETs in pregnancy complications. NETs play pivotal roles in the pathogenesis of various pregnancy complications: **(a)** in PE, STBM, IL-8 and sFlt-1 drive elevated NETosis, triggering inflammation, endothelial injury, spiral artery remodeling disorder and placental infarction; **(b)** in GDM, increased TNFα levels, reduced adiponectin levels and hyperglycemia-induced PAD4 activation promote NET overproduction, which impairs trophoblast proliferation, invasion and glucose metabolism; **(c)** in PTB, intrauterine infection and TNFα induce NET formation via MAPK/ROS pathways, leading to amniotic epithelial cell apoptosis and premature rupture of membranes; **(d)** in RPL, a dysregulated percentage of LDGs and sFlt-1-mediated NET accumulation cause recurrent embryo loss; **(e)** in SLE with APOs, impaired DNase-mediated NET clearance and elevated LDGs cause NET accumulation, which induces trophoblast pyroptosis and alters dNK subsets to worsen placental injury. **(f)** in OAPS, aPLs induce NET formation via TLR4, impairing trophoblast and endothelial function through multiple pathways to cause placental injury and APOs. Figure created with BioRender.com.

This section sequentially discusses the specific molecular mechanisms, clinical manifestations, and existing evidence of NET involvement in various obstetric complications (**Figure 3**).

### 4.1 Preeclampsia

PE is a pregnancy-specific condition categorized among the hypertensive disorders of pregnancy, which typically manifests after 20 weeks of gestation. Its core characteristics include *de novo* hypertension and proteinuria or the development of other organ damage in the absence of proteinuria (93). This condition poses a severe threat to maternal and fetal health and constitutes one of the leading causes of maternal and perinatal morbidity and mortality. To date, termination of pregnancy remains the only effective curative intervention (94).

In patients with PE, neutrophil counts increase significantly as early as the second trimester, and they are correlated with disease severity (95). Notably, compared with those with normal pregnancies, those with PE exhibit a marked increase in NET formation via the non-lytic pathway (9). Moreover, neutrophils in the maternal circulation and placental tissues of PE patients exhibit excessive activation, accompanied by increased NET formation (13, 96).

The underlying mechanisms contributing to this phenomenon are multifaceted. Reports have indicated that BCL2-related protein A1 (BCL2A1, an anti-apoptotic BCL-2 family protein) and G0/G1 switch gene 2 (G0S2, a lipid metabolism-and apoptosis-related protein) are overexpressed in neutrophils within the placental tissues of PE patients and that overexpression of these two molecules promotes NET release from neutrophils (97). Additional studies have suggested that placenta-derived syncytiotrophoblast microparticles (STBMs) and interleukin-8 (IL-8) in PE patients can activate neutrophils and subsequently induce NETosis in a dose-dependent manner (33, 98). Furthermore, these findings revealed that phospholipase A/acyltransferase 3 (PLAAT3, a phospholipid-metabolizing enzyme) expression is upregulated in the syncytiotrophoblasts (STB) of PE placentas, and this upregulated PLAAT3 activates neutrophils via the LPA-LPAR5 axis (lysophosphatidic acid and its receptor 5 signaling pathway), thereby leading to NET release (99). In addition, the level of soluble fms-like tyrosine kinase 1 (sFlt-1), an anti-angiogenic protein, is elevated in the serum of PE patients (100), and sFlt-1 has been shown to induce neutrophils to release NETs (101). However, it has been reported that in some special clinical scenarios, such as pregnant women with PE complicated by human immunodeficiency virus (HIV) infection, placental NET infiltration is conversely reduced. This phenomenon may be explained by the HIV-triggered release of the anti-inflammatory cytokine interleukin-10 from DCs in affected individuals, which subsequently inhibits the formation of NETs (102–104).

NETs contribute to the pathogenesis and progression of PE through multiple pathophysiological pathways. NETs induce oxidative stress, apoptosis, and dysfunction in human umbilical vein endothelial cells (HUVECs) and trophoblasts (105). Sera from PE patients can promote NET formation, which activates complement activity and further aggravates placental endothelial injury (16). In addition, NETs suppress trophoblast function via their component high mobility group B1 (HMGB1) and promote the release of ROS and proinflammatory cytokines (e.g., IL-1β, IL-6, IL-8, and TNF-α), thereby exacerbating placental inflammation and tissue injury (15). The combined effects of endothelial damage, trophoblast dysfunction, and the procoagulant activity of NETs ultimately impair spiral artery remodeling, disrupt placental vascular development, and contribute to placental injury, infarction, and APOs (1, 106, 107).

### 4.2 Gestational diabetes mellitus

GDM is traditionally defined as the onset or first identification of impaired glucose tolerance during pregnancy. This condition has long been associated with various obstetric and neonatal complications, particularly fetal macrosomia, and it is increasingly recognized as a significant risk factor for future cardiometabolic diseases in both mothers and their offspring (108). The pathogenesis of GDM has not yet been fully elucidated, but dysregulated immune modulation is considered one of its key mechanisms (109).

Increasing evidence supports a central role for NETs in GDM pathophysiology. The neutrophil-to-lymphocyte ratio (NLR) is significantly increased in the serum of patients with GDM (110). Peripheral blood neutrophils from GDM patients exhibit enhanced activation and a greater propensity to form NETs *in vitro*, which may be driven by elevated circulating TNF-α levels (111). Compared with healthy pregnant women, pregnant women with GDM are characterized by greater spontaneous NET formation and lower serum adiponectin levels. Since adiponectin normally attenuates NET release by inhibiting ROS production, its reduction may contribute to the increased NETs observed in GDM (112). Furthermore, hyperglycemia, a common feature of GDM, has been reported to promote NET formation by modulating PAD4 activity (113, 114).

Notably, elevated NETs are detected in both the peripheral circulation and placental tissues of women with GDM, and these excessive NETs directly impair the proliferation, invasion, migration, and angiogenic capacity of placental trophoblasts, indicating that NETs contribute to the pathogenesis of GDM by disrupting trophoblast function. Importantly, the choline metabolite trimethylamine N-oxide (TMAO) can alleviate these deleterious effects by suppressing NET formation. Consistent with these findings, dietary TMAO supplementation in GDM mouse models reduces systemic NET generation and improves placental and fetal development, further supporting the involvement of NETs in GDM (10). In addition to direct structural and functional damage, neutrophil-derived NE impairs trophoblast physiology and glucose metabolism by downregulating the expression of the glucose transporter GLUT4 (115). Moreover, NETs amplify placental injury in GDM by inducing trophoblast apoptosis through activation of the ROS-dependent mitochondrial pathway and inhibiting of the pro-survival ERK1/2 signaling pathway (112).

Taken together, these findings demonstrate that multiple pathological triggers of GDM, including inflammation, low adiponectin levels, and hyperglycemia, converge to promote excessive NET formation, which in turn leads to impaired trophoblast function, disrupted glucose metabolism, and enhanced placental damage, thereby contributing to the development and progression of GDM.

### 4.3 Preterm birth

PTB is defined as delivery occurring before 37 weeks of gestation (116). The majority of preterm births are spontaneous, arising from two primary pathways: (1) spontaneous labor with intact membranes and (2) preterm premature rupture of membranes (117). The precise pathogenesis of PTB remains incompletely understood, and intrauterine infection is a well-recognized key trigger, accounting for approximately 40% of spontaneous PTB cases (116, 118). Chorioamnionitis, the typical pathological manifestation of intrauterine infection, is characterized by massive neutrophil infiltration and a robust inflammatory response at the maternal-fetal interface (119, 120), and accumulating evidence has confirmed the close association between NETs and the occurrence of PTB.

Clinically, the peripheral blood neutrophil count and NLR are significantly increased in women with PTB and can serve as potential predictive biomarkers for PTB (121). In contrast to neutrophils from the peripheral blood of normal pregnancies, the amniotic fluid neutrophils of patients with PTB are capable of spontaneous NET formation (122). Notably, elevated neutrophil levels and substantial NET formation have been detected in the placental membranes, amniotic fluid and amnion of preterm patients with acute chorioamnionitis (123–125). Lipopolysaccharide, from gram-negative bacteria is a common microbial stimulant in intrauterine infection, and group B Streptococcus is a pathogenic bacterium that can cause intrauterine infection complicated by preterm birth; both induce NET formation at the maternal-fetal interface (11, 126, 127). Fetal membrane tissues secrete a variety of proinflammatory cytokines (IL-1β, IL-6, IL-8 and TNF-α) in response to microbial stimulation (128), among which IL-8 specifically recruits peripheral neutrophils to the maternal-fetal interface (129), and TNF-α further activates neutrophils and promotes NET formation through the mitogen-activated protein kinase (MAPK) signaling pathway to induce ROS production (11).

NETs can activate the ERK1/2 signaling pathway to promote ROS generation, which in turn induces the apoptosis of human amniotic epithelial cells and further aggravates fetal membrane damage and PTB progression (125). In addition, activated neutrophils have been shown to reduce chorioamniotic membrane integrity and tensile strength (130). As NETs contain hydrolytic enzymes such as NE capable of degrading collagen fibers, the mechanism by which NETs may contribute to PTB may be attributable to a reduction in fetal membrane elasticity and mechanical strength. Further research is needed to verify this mechanism.

### 4.4 Recurrent pregnancy loss

RPL is clinically defined as the occurrence of two or more consecutive pregnancy losses prior to 20–24 weeks of gestation, encompassing both embryonic loss and fetal death (131). While the exact etiology of RPL has not been fully elucidated, current studies suggest that dysregulated immune modulation at the maternal-fetal interface, particularly aberrant neutrophil activation and NET formation, may be closely associated with the pathogenesis of some RPL cases (132).

Compared with healthy pregnant women, women with RPL have a significantly elevated NLR in serum, suggesting that NLR may serve as a potential early auxiliary diagnostic marker for RPL (133). There is direct evidence indicating the increased formation of NETs in the serum and decidual tissues of patients with RPL (134), which points to a close association between excessive NET generation and pathogenesis of RPL. Notably, RPL represents repeated episodes of spontaneous abortion (SA) (135), and the pathological mechanisms underlying SA constitute the fundamental basis for recurrent miscarriage. Therefore, relevant evidence from SA is highly informative for understanding RPL. A dysregulated percentage of LDGs was found in the decidual tissues of patients with SA (12). LDGs isolated from the decidual tissues of SA patients show increased cellular activation, as evidenced by the elevated expression of CD11b, a well-established marker of neutrophil activation. Moreover, extensive NET infiltration is also detected in the decidual tissues of SA patients. These findings imply that LDGs and the NETs they form may contribute to the pathogenesis of SA (12), which may further contribute to the recurrent pregnancy loss. Overexpression of sFlt-1 induces significant pregnancy loss and massive NET accumulation in mouse placentas. Notably, gene knockout of PAD4 in mice markedly ameliorated the APOs such as miscarriage induced by sFlt-1 (101). These results further suggest that excessive NET formation may be a common pathological driver of pregnancy loss, which is highly relevant to RPL given its recurrent nature.

Studies directly investigating the role of NETs in RPL are relatively scarce. Nevertheless, the above evidence suggests that NETs contribute to pregnancy loss in single miscarriages and that repetitive activation of this mechanism may underlie the recurrent phenotype of RPL.

Although evidence has confirmed an association between NETs and RPL such as the abnormal accumulation of NETs in serum and decidual tissues direct causal evidence linking NETs to the pathogenesis of RPL is still lacking, and the specific molecular mechanisms by which NETs induce pregnancy loss remain unclear.

### 4.5 Autoimmune diseases

#### 4.5.1 Systemic lupus erythematosus

SLE is a chronic autoimmune disease characterized by systemic inflammation and immune-mediated damage affecting multiple organ systems, frequently involving the mucocutaneous, musculoskeletal, hematological, and renal systems (136). SLE affects women in approximately 90% of cases, and these patients face a significantly elevated risk of APOs, including miscarriage, PE, PTB, and fetal growth restriction (137). Placental injury resulting from dysregulated immune adaptation at the maternal-fetal interface is a key mechanism underlying APOs in patients with SLE (138). Recent studies have demonstrated that patients with SLE complicated by APOs exhibit an elevated serum NLR, suggesting that the NLR may serve as a potential biomarker for predicting the risk of APOs in patients with SLE (139, 140). Additionally, the proportion of LDGs is greater in the peripheral blood of pregnant women with SLE than in those with healthy pregnancies (141, 142). Moreover, studies in patients with SLE have demonstrated reduced secretion and decreased activity of DNase1 and DNase1L3, leading to impaired NET clearance (143, 202). Although these findings were primarily obtained in non-pregnant individuals, they provide a mechanistic basis for understanding excessive NET accumulation in patients with SLE with APOs.

High levels of NET infiltration have been observed in the intervillous spaces of placental villi from SLE patients with APOs, indicating a potential role for NETs in the pathogenesis of APOs in this patient population (13). Further mechanistic studies revealed that NETs can promote glycolysis in trophoblasts and increase lactylation (a novel post-translational modification driven by lactate) of the NLRP3 inflammasome, thereby activating the NLRP3 inflammasome and inducing trophoblast pyroptosis, ultimately leading to placental damage. Consistently, in SLE mouse models, blocking NETs either by NET degradation with DNase1 or by inhibiting NET formation via PAD4 knockout has been shown to attenuate these pathological processes and reduce APOs (144). Decidual natural killer cells (dNKs) contribute to the maintenance of maternal-fetal interface immune tolerance, the promotion of trophoblast invasion, and the remodeling of spiral arteries through the secretion of cytokines and chemokines (145). Alterations in the number and functional activity of dNK cells during pregnancy are closely associated with the development of APOs (146). In SLE patients with APOs, dNKs are significantly increased in the placental intervillous space, with a notable increase in the level of cytotoxic CD56^+^CD16^+^ NK cell subset. Moreover, the extent of NET infiltration is positively correlated with the number and subtype distribution of dNKs. The inhibition of NET formation attenuates the aberrant infiltration of dNKs, suggesting that NETs may contribute to placental dysfunction in patients with SLE by modulating the subset composition and quantity of dNKs (147).

#### 4.5.2 Antiphospholipid syndrome

APS is an autoimmune disorder mediated by antiphospholipid antibodies (aPLs) and is characterized by thrombosis and APOs (148). Based on its primary clinical manifestations, APS can be categorized into two distinct subtypes: thrombotic APS (TAPS), which is dominated by thrombotic complications, and obstetric APS (OAPS), which is defined by pregnancy complications. Typical features of OAPS include RPL, unexplained fetal death beyond 10 weeks of gestation, early-onset PE, and PTB due to placental dysfunction (149). aPLs mediate pregnancy complications by initiating the complement cascade, and elevated levels of local complement activation fragments have severe detrimental effects on fetal development (150). Furthermore, circulating NET levels are found to be elevated in patients with APS. In *in vitro* assays, aPLs have been demonstrated to activate neutrophils via Toll-like receptor 4 (TLR4), thereby promoting ROS production and inducing NET formation (37).

The underlying mechanisms of placental injury in OAPS have not been fully elucidated. In recent years, studies from our research group have demonstrated that NETs play a pivotal role in the pathogenesis and progression of OAPS. First, our findings revealed that pregnant women with APS exhibit elevated serum NET levels, and neutrophils isolated from their peripheral blood show an enhanced spontaneous ability to release NETs *in vitro*. Further experimental evidence has confirmed that purified aPLs from the peripheral blood of pregnant women with APS can directly induce NETosis, and that these NETs inhibit the functional activity of both trophoblasts and HUVECs (14). Second, increased NET infiltration has been observed in placental tissues from both OAPS patients and corresponding mouse models. Mechanistically, NETs promote the generation of ROS in trophoblasts, which activates BNIP3 (a key mitophagy regulator)-mediated mitophagy and subsequently induces trophoblast apoptosis, thereby exacerbating placental damage (151). In addition, NETs can also trigger trophoblast pyroptosis via the MAPK and NF-κB signaling pathways, leading to further impairment of placental function. Notably, HCQ administration effectively alleviated NET-induced trophoblast injury (152). Taken together, these findings highlight the critical role of NETs in aPL-mediated placental pathology and associated APOs.

## 5 Pharmacological modulation of NETs in obstetrics

Given the critical role of NETs in the pathophysiology of a wide range of pregnancy-related complications, targeted regulation of NET formation has become a highly promising therapeutic approach. Notably, several drugs already employed in the clinical management of these complications have recently been shown to modulate NET formation to varying extents, revealing a previously unrecognized dimension of their pharmacological activity (**Table 1**).

**Table 1:** Obstetric drugs targeting NETs formation.

Drugs	Indication	Mechanism of action	Human evidence (in vitro/ vivo)	Aniaml model evidence	Reference
Aspirin	Preeclampsia OAPS	Inhibits platelet activation and TXA_2_ production, which in turn inhibits neutrophil activation and subsequent NET formation.	1. Inhibits NET formation in human neutrophils stimulated by COVID-19 patient serum 2. Blocks platelet-human neutrophil interaction and subsequent NET formation	1. Reduces NET formation and venous thrombosis in mouse thrombosis models 2. Alleviates lung injury by inhibiting NET formation in mouse transfusion/PGD models	(17, 158–164)
Metformin	GDM	Modulates NET formation either by inhibiting NADPH oxidase activation or by enhancing the phagocytic capacity of macrophages.	1. Reduces NET accumulation in human CRC tissues of T2DM patients 2. Enhances macrophage-mediated NET clearance in ARDS patients 2. Inhibits high glucose/PMA/HMGB1-induced NETosis in human neutrophils	Inhibits adipocyte-induced NET formation in obese mouse pancreatic tissue	(18, 85, 167–172)
LMWH	RPL OAPS	1. Directly inhibits neutrophil activation and NET formation. 2. Alleviates tissue damage by binding to histones and mitigating their cytotoxicity.	1. Reduces NET release from human neutrophils stimulated by COVID-19 patient serum 2. Inhibits pro-thrombotic stimulus-induced NETosis in human neutrophils	1. Reduces NET accumulation and lung injury in COVID-19 mouse models 2. Improves survival in mouse models of histone-induced organ dysfunction 3. Attenuates rat histone-mediated endothelial cells injury by histone binding	(17, 175, 176, 179, 180)
HCQ	SLE with APOs OAPS	1. Inhibits NET formation through suppressing PAD4 enzymatic activity and blocking TLR9 signaling pathway 2. Inhibits PRL2 degradation, regulating Rac GTPase/ROS-mediated NETosis	Reduces spontaneous NET formation in peripheral blood neutrophils of healthy donors	1. Alleviates liver ischemia/reperfusion injury by inhibiting NET formation in mice 2. Ameliorates NET-induced tissue damage in malaria and acute lung injury model mouse models 3. Inhibits mouse neutrophil NETosis via TLR9 pathway	(20, 183–187)
Vitamin D	VitaminD deficiency	1. Inhibits *NET-forming capacity* of neutrophil activation and NET formation 2. Synergizes with ω3 PUFAs to inhibit NETosis	1. Reduces PMA-induced NET formation in SLE patient neutrophils 3. Combined with ω3 PUFAs exerts inhibit the ability of neutrophils to generate NETs in T2DM patient	1. Suppresses NET formation and ameliorates alveolarization disorders in hyperoxia-induced BPD rat models 2. Mitigates NET-induced ovarian fibrosis in premature ovarian insufficiency mouse models	(21, 195–197, 200, 201)

### 5.1 Aspirin

Aspirin is clinically used for the prevention of PE and the management of OAPS (153–155). As a nonsteroidal anti-inflammatory drug, aspirin exerts its antithrombotic effect primarily by inhibiting cyclooxygenase activity, thereby reducing the synthesis of thromboxane A_2_ (TXA_2_) (156). TXA_2_ is a potent vasoconstrictor that activates and promotes platelet aggregation (157). In addition, activated platelets in the circulation not only contribute to thrombogenesis but also stimulate NET formation through the release of HMGB1, which engages receptors for advanced glycation end products and TLR4 on neutrophils (158, 159). Given the regulatory role of platelets in neutrophil activation and NET formation, antiplatelet agents such as aspirin may reduce NET formation by targeting platelet function.

Multiple experimental studies have provided evidence in support of this mechanism. For instance, serum from COVID-19 patients can induce robust NETosis and NET formation in neutrophils, and these effects can be significantly suppressed by aspirin (17). *In vitro* assays have further revealed that platelets activated by intrahepatic cholangiocarcinoma cells via P-selectin can promote neutrophil NET release and cancer cell migration, both of which are inhibited by aspirin treatment (160). Similarly, a study by Lapponi et al. demonstrated that aspirin, but not the steroid immunomodulatory drug dexamethasone, effectively suppresses NET formation (161). In a mouse model of venous thrombosis, aspirin exerts its antithrombotic effects by inhibiting platelet-derived TXA_2_ production, which in turn suppresses neutrophil activation and subsequent NET formation (162). Moreover, in murine models of lung injury and primary graft dysfunction, aspirin attenuates lung tissue damage by inhibiting platelet activation and subsequent NET generation (163, 164).

### 5.2 Metformin

Metformin is a hypoglycemic agent clinically indicated for the management of GDM (165). Its primary pharmacological action is mediated by the inhibition of mitochondrial complex I activity, which subsequently activates AMP-activated protein kinase (AMPK) and suppresses hepatic gluconeogenesis, ultimately exerting a glucose-lowering effect (166). In recent years, emerging evidence has revealed that metformin plays a regulatory role in the formation of NETs. In patients with type 2 diabetes mellitus, metformin administration has been shown to reduce the enrichment of tumor-associated neutrophils and NETs in colorectal cancer tissues (18). In obese mouse models, metformin decreases neutrophil infiltration in pancreatic tissue and inhibits adipocyte-induced NET formation (167). Further *in vitro* cellular assays have demonstrated that metformin is capable of suppressing NET formation induced by various stimuli, including high glucose, PMA, and HMGB1 (85, 168–170).

These findings collectively indicate that metformin participates in the regulation of NET formation, although the underlying mechanisms remain incompletely elucidated. It has been reported that metformin can inhibit the activation of NADPH oxidase by preventing the translocation of PKC from the cytoplasm to the cell membrane, thereby interfering with NETosis (171). In addition, metformin can activate AMPK signaling pathway in macrophages, increasing their ability to phagocytose NETs and consequently reducing NET accumulation in acute respiratory distress syndrome (ARDS) patients (172).

### 5.3 Low molecular weight heparin

LMWH is a commonly used agent for improving APOs, such as RPL, in patients with OAPS (173). As a depolymerized fragment derived from unfractionated heparin via chemical or enzymatic approaches, LMWH is widely employed in clinical anticoagulant therapy (174). LMWH can inhibit neutrophil activation, autophagy, and the capacity to form NETs (19). LMWH reduces NETs levels in neutrophils stimulated with COVID-19 patient serum (17). Furthermore, LMWH reduces NET accumulation and alleviates lung injury in COVID-19 animal models (175, 176). In addition to its direct inhibitory effects on NET generation, LMWH can also antagonize the pathological effects of histones, which are key structural components of NETs. Histones can activate the NF-κB signaling pathway to promote cytokine release, thereby amplifying inflammatory responses and inducing organ damage (177–179). Both *in vitro* and *in vivo* experiments have verified that heparin can bind to histones, attenuate their cytotoxicity, mitigate subsequent tissue injury, and protect mice from histone-mediated organ dysfunction and even mortality (180).

### 5.4 Hydroxychloroquine

HCQ is a commonly used agent in the management of SLE with obstetric complications and OAPS (181). Initially introduced into clinical practice as an antimalarial drug, HCQ was subsequently found to possess potent immunomodulatory and anti-inflammatory properties during long-term clinical application, which has led to its progressive repurposing for the treatment of a broad spectrum of autoimmune disorders, including SLE, APS, RA, and primary Sjögren’s syndrome (182). Findings from recent investigations have demonstrated that HCQ inhibits NET formation through multiple mechanisms. *In vitro* experiments have confirmed the inhibitory effect of HCQ on NET formation (183–185), a mechanism potentially attributed to its ability to regulate PAD4 either by inhibiting its enzymatic activity or by suppressing its expression by blocking the Toll-like receptor 9 (TLR9) signaling pathway (20, 186). In a murine model of hepatic ischemia/reperfusion injury, HCQ can alleviate liver tissue damage by inhibiting NET formation (20). Additionally, in mouse malaria and acute lung injury models, HCQ also participates in the regulation of NET formation by inhibiting the degradation of phosphatase of regenerating liver 2, which negatively regulates NETosis by modulating Rac GTPase activity and ROS production (187).

### 5.5 Vitamin D

Vitamin D, a fat-soluble secosteroid hormone, plays a critical role in maintaining homeostasis across multiple organ systems (188). Vitamin D deficiency is commonly observed in individuals with inadequate sun exposure, those with darker skin pigmentation, and pregnant women (189). Vitamin D deficiency is closely associated with various APOs, such as hypertensive disorders of pregnancy, GDM, PTB, and being small for gestational age (190–193). Therefore, timely vitamin D supplementation in deficient pregnant women is recommended to mitigate some of these risks (194).

Vitamin D supplementation has been shown to reduce the incidence of PE and GDM (195), promote the release of proinflammatory cytokines (IL-1β and IL-8) from neutrophils isolated from the peripheral blood of healthy people (196), and simultaneously inhibit the formation of NETs-an effect that is more pronounced when combined with ω-3 polyunsaturated fatty acids (PUFAs) (197). However, two clinical *in vivo* studies in healthy Saudi populations (198, 199) confirmed that a single high-dose (80,000 IU) vitamin D_3_ bolus significantly reduced serum IL-6, IL-8, and TNF-α levels without disrupting calcium-phosphorus homeostasis. This discrepancy between *in vitro* and *in vivo* findings may be because vitamin D modulates the entire immune system to exert systemic anti-inflammatory effects *in vivo*, which is distinct from its effect on isolated cells. Vitamin D supplementation significantly alleviates NET-induced early apoptosis of HUVECs by reducing NET release from the neutrophils of vitamin D-deficient SLE patients (200). In the field of reproductive health, vitamin D can mitigate NET-induced oxidative stress and fibrosis in ovarian tissues by inhibiting NET formation, thereby delaying the onset of premature ovarian insufficiency (201). In addition, vitamin D supplementation increases the survival rate of rats with hyperoxia-induced bronchopulmonary dysplasia and ameliorates alveolarization disorders by suppressing NET formation (21). Notably, vitamin D supplementation may regulate the NET-forming capacity of neutrophils by altering the systemic proinflammatory cytokine profile. Changes in cytokine status serve as an important intermediate links connecting vitamin D levels and NET formation and are key confounding factors. Currently, the direct molecular mechanisms by which vitamin D regulates NET formation (e.g., whether it directly targets key molecules involved in NET formation, such as PAD4 or NADPH oxidase) remain to be verified by more in-depth mechanistic experiments.

Although aspirin, metformin, LMWH, HCQ and vitamin D have been shown to regulate NET formation or counteract its pathological effects through distinct pharmacological approaches, direct experimental or clinical evidence verifying the specific regulatory effects of these drugs on NETs in obstetric complications remains insufficient to varying degrees. Collectively, the aforementioned findings indicate that targeting the homeostasis of NETs is a promising potential therapeutic strategy for pregnancy complications, and further in-depth clinical and basic research is needed to clarify the specific regulatory mechanisms of these clinical obstetric drugs on NETs during pregnancy to provide a more solid theoretical basis for their clinical application in preventing and treating pregnancy complications mediated by the imbalance of NETs.

## 6 Conclusion and outlook

While NETs play a beneficial role in host defense by trapping and eliminating pathogens, the concomitant release of proteolytic enzymes and cytotoxic proteins can cause tissue damage, thereby implicating NETs in the pathogenesis of various diseases. As a result, the regulation of NET formation is increasingly being explored as a therapeutic target for multiple disorders. This review systematically examines the dual functions of NETs in normal pregnancy and in obstetric complications such as PE, GDM, PTB, and RPL, along with their pathological roles in pregnancies complicated by autoimmune diseases, including APS and SLE. Growing evidence suggests that as key elements of innate immunity, NETs play a protective physiological role at the maternal-fetal interface, helping to maintain the immune microenvironment and defend against infections. However, excessive NET formation or inadequate clearance may contribute to placental dysfunction and pregnancy-related pathology through various mechanisms, such as impaired trophoblast function, induction of a prothrombotic state, and amplification of inflammatory responses.

This review also synthesizes emerging evidence on how common obstetric medications, such as aspirin, metformin, LMWH, HCQ, and vitamin D, may exert therapeutic benefits. These findings suggest that the positive effects of these compounds on pregnancy outcomes could be partly mediated through the modulation of NET formation and clearance. These findings expand the understanding of the pharmacological mechanisms of existing drugs and offer new insights for optimizing clinical treatment strategies.

Despite the significant progress of current research, substantial limitations remain. Most studies have focused on animal models and *in vitro* experiments, while research on human pregnancy, particularly across different gestational stages, remains insufficient. Notably, the regulatory effects of pregnancy-related hormones on NET formation and homeostasis in pregnancy complications remain to be fully elucidated because of the lack of definitive experimental evidence, which is a critical research gap in the field of NET and pregnancy-related disorder research. Furthermore, the absence of standardized methods for the detection and quantification of NETs *in vivo* hinders their development as clinical diagnostic markers. Additionally, the long-term safety and efficacy of NET-targeted therapies in pregnancy have not been fully validated.

Based on the existing research foundation, future research directions can focus on the following aspects. First, a standardized NET detection system that integrates imaging and molecular biology techniques to enable precise monitoring of NET levels throughout pregnancy should be established, with the aim of identifying specific biomarkers for early warning of pregnancy complications. Second, large-sample, multi-center longitudinal clinical studies should be conducted to elucidate the regulatory dynamics of NETs across gestational ages, clarify the correlation between disrupted NET homeostasis and the severity of pregnancy complications, and provide a basis for stratified disease management. Third, the development of NET-targeted therapies, such as optimizing the administration regimen of DNase1 preparations and developing specific PAD4 inhibitors, while exploring combination strategies to enhance efficacy and reduce potential risks to the mother and fetus. Fourth, integrate multi-omics technologies should be integrated to explore the interactions between NETs and immune cells and cytokine networks at the maternal-fetal interface, thereby revealing the molecular pathways through which NETs regulate pregnancy outcomes from a systemic perspective. Fifth, there should be a focus on special populations, such as pregnant individuals with HIV infection, obesity, or other high-risk factors, to analyze their NET metabolic profiles and develop individualized intervention strategies.

In summary, this review systematically elucidates the central role of NET homeostasis imbalance in pregnancy complications and emphasizes the significance of targeted NET regulation for improving maternal and fetal outcomes. With further research, NETs are expected to become key targets for the precise diagnosis and treatment of pregnancy complications, offering novel breakthroughs in the prevention and management of obstetric disorders.
